# Comparison of the Ki-67 score and S-phase fraction as prognostic variables in soft-tissue sarcoma

**DOI:** 10.1038/sj.bjc.6690151

**Published:** 1999-02

**Authors:** R L Huuhtanen, C P Blomqvist, T A Wiklund, T O Böhling, M J Virolainen, E J Tukiainen, B Tribukait, L C Andersson

**Affiliations:** Department of Oncology, Helsinki University Central Hospital, Helsinki, FIN-00029-HYKS, Finland; Department of Pathology, University of Helsinki, Haartman Institute, S-17176-Stockholm, Sweden; Helsinki University Central Hospital, Department of Plastic Surgery, S-17176-Stockholm, Sweden;; Karolinska Institute and Hospital,Department of Radiology, S-17176-Stockholm, Sweden

**Keywords:** soft-tissue sarcoma, prognosis, S-phase, Ki-67, flow cytometry, immunohistochemistry

## Abstract

Immunohistochemically determined Ki-67 scores and flow cytometrically determined S-phase fractions were successfully evaluated from the primary tumours of 123 patients with soft-tissue sarcoma. All patients had either limb or superficial trunk tumours. Ki-67 score correlated strongly with ploidy, S-phase fraction and grade. Ki-67 did not correlate with the size of the primary tumour. When analysed as a continuous variable, Ki-67 was a stronger predictor of both metastasis-free survival and disease-specific overall survival (*P*= 0.003 and 0.04 respectively) than was the S-phase fraction (*P*= 0.06 and 0.07 respectively). We tested the relevance of different cut-point values by dividing the whole material into two parts at every 10% (e.g. 10% of patients vs. the remaining 90%, 20% vs. 80%, etc.). We counted the relative risk and confidence interval at all these cut-off points. Ki-67 had good prognostic discriminating power irrespective of the cut-point value, but S-phase fraction lost its prognostic power at higher cut-point values. In conclusion, we found that Ki-67 is a useful prognostic tool in the treatment of soft-tissue sarcoma patients irrespective of the cut-point value. S-phase fraction can be used at lower cut-point values. *© 1999 Cancer Research Campaign*


*British Journal of Cancer* (1999) **79**(5/6), 945–951
© 1999 Cancer Research Campaign
Article no. bjoc.1998.0151

### Comparison of the Ki-67 score and S-phase fraction as prognostic variables in soft-tissue sarcoma

**RL Huuhtanen[1], CP Blomqvist[1], TA Wiklund[1], TO Böhling[2], MJ Virolainen[2], EJ Tukiainen[3], B Tribukait[4] and LC Andersson[2]**

[1]Helsinki University Central Hospital, Department of Oncology, FIN-00029-HYKS, Finland; [2]University of Helsinki, Haartman Institute, Department of Pathology, S-17176-Stockholm, Sweden; [3]Helsinki University Central Hospital, Department of Plastic Surgery, S-17176-Stockholm, Sweden; [4]Karolinska Institute and Hospital, Department of Radiology, S-17176-Stockholm, Sweden

**Summary** Immunohistochemically determined Ki-67 scores and flow cytometrically determined S-phase fractions were successfully evaluated from the primary tumours of 123 patients with soft-tissue sarcoma. All patients had either limb or superficial trunk tumours. Ki-67 score correlated strongly with ploidy, S-phase fraction and grade. Ki-67 did not correlate with the size of the primary tumour. When analysed as a continuous variable, Ki-67 was a stronger predictor of both metastasis-free survival and disease-specific overall survival ($P = 0.003$ and 0.04 respectively) than was the S-phase fraction ($P = 0.06$ and 0.07 respectively). We tested the relevance of different cut-point values by dividing the whole material into two parts at every 10% (e.g. 10% of patients vs. the remaining 90%, 20% vs. 80%, etc.). We counted the relative risk and confidence interval at all these cut-off points. Ki-67 had good prognostic discriminating power irrespective of the cut-point value, but S-phase fraction lost its prognostic power at higher cut-point values. In conclusion, we found that Ki-67 is a useful prognostic tool in the treatment of soft-tissue sarcoma patients irrespective of the cut-point value. S-phase fraction can be used at lower cut-point values.

**Keywords:** soft-tissue sarcoma; prognosis; S-phase; Ki-67; flow cytometry; immunohistochemistry

A high proliferation rate predicts a poor prognosis in many types of malignancies. Several methods are available for assessing proliferation in human tumours (Keshgegian and Cnaan, 1995). The two most common methods used in recent years have been flow cytometric measurement of the S-phase fraction (SPF) and immunohistochemical staining of the proliferation antigen Ki-67. The Ki-67 antigen is expressed over the whole active cell cycle phases from G1 to M (Gerdes et al, 1991).

Many studies indicate that in breast cancer proliferation activity measured by either Ki-67 antibody or by flow cytometric SPF has prognostic value (Joensuu et al, 1990; Sigurdsson et al, 1990; Weikel et al, 1991, 1995; Railo et al, 1993; Veronese et al, 1993; Camplejohn et al, 1995; Dettmar et al, 1997; Molino et al, 1997).

Few studies, however, have compared the prognostic value of these two methods. The results of such studies have been variable as regards breast cancer (Gasparini et al, 1994; Keshgegian and Cnaan, 1995; Pierga et al, 1996; Dettmar et al, 1997). In a recent extensive study, Ki-67 was found to be a stronger prognostic factor than SPF (Railo et al, 1997).

Several studies on soft-tissue sarcomas (STS) have shown that a high expression of Ki-67 predicts an adverse prognosis. Most of these studies are based on a limited number of cases, and few have included more than 100 patients (Choong et al, 1994; Drobnjak et al, 1994; Rudolph et al, 1997).

We have found previously that a high SPF was associated with poor survival in patients whose primary tumour was diploid. SPF did not predict survival in patients with non-diploid tumours (Huuhtanen et al, 1996). Other studies on the prognostic value of SPF have been inconsistent, although two recent large studies suggest that SPF has prognostic value irrespective of ploidy when a low cut-point value of 3% or 4% is used (Collin et al, 1997; Gustafson et al, 1997).

The aim of this study was to evaluate the prognostic value of Ki-67 in STS and to compare it with SPF.

*Received 4 February 1998*
*Revised 18 May 1998*
*Accepted 29 May 1998*

*Correspondence to:* R Huuhtanen, Helsinki University Central Hospital, Department of Oncology, PO Box 180, FIN-00029-HYKS, Finland

### PATIENTS AND METHODS

### Patients

The patients were consecutive adult patients treated for STS of the limbs or superficial trunk by the soft-tissue sarcoma group at Helsinki University Central Hospital between January 1987 and May 1993. They were treated according to the guidelines of the Helsinki STS group (Wiklund et al, 1996). In the present prognostic study, we included patients with primary tumour and SPF determined in the context of our previous study, with the exception of patients with dermatofibrosarcoma protuberans (Huuhtanen et al, 1996).

The number of patients was 145. Formalin-fixed, paraffin-embedded histological samples were available for immunohistochemical staining in 135 cases. In 12 cases, the Ki-67 staining was repeatedly unsuccessful, probably because of inappropriate sample fixation. Histopathological subtypes and grades of the missing samples, and the samples with unsuccessful Ki-67 staining are shown in Table 1.

Altogether 123 patients were finally included in this study. One patient had a tumour which was limited to the skin only. Thirty-eight tumours were subcutaneous, or both subcutaneous and cutaneous. In 82 cases, the primary tumour was located deep within

**Table 1** Histology and grade of the missing samples and the unsuccessful Ki-67 stainings

| Histology | Missing samples, *n* | Unsuccessful staining, *n* |
|---|---|---|
| Malignant fibrous histiocytoma | 4 (G3 = 3, G4 = 1)[a] | 1 (G4 = 1) |
| Leiomyosarcoma | 2 (G1 = 1, G3 = 1) | 4 (G1 = 1, G3 = 2, G4 = 1) |
| Synovial sarcoma | 1 (G3 = 1) | 1 (G3 = 1) |
| Extraskeletal Ewing's sarcoma | 2 (G4 = 2) | |
| Epithelioid sarcoma | 1 (G3 = 1) | |
| Liposarcoma | | 2 (G1 = 2) |
| Extraskeletal osteosarcoma | | 2 (G3 = 1, G4 = 1) |
| Unclassified sarcoma | | 1 (G4 = 1) |
| Fibrosarcoma | | 1 (G1 = 1) |

[a]G1–G4 = number of patients with grade 1–4 tumours.

the tissue. In two cases, information on the site was missing. The pretreatment characteristics of the patients in this study are shown in Table 2.

The Helsinki STS group is a member of the Scandinavian Sarcoma Group (SSG), in which a four-grade histological malignancy grading system is applied. For the grading, the combination of the histological type and a combination of histopathological parameters, which include necrosis, vascular invasion, cellularity, mitotic activity and nuclear pleomorphism, is used. Since the publication of our study on SPF (Huuhtanen et al, 1996), the histopathological subtypes have been changed in seven cases during the peer review sessions by the SSG pathologists.

The median follow-up time of living patients ($n$ = 71) was 72 months (range 22–447 months). Four patients have died without disease. Fifty-one patients developed metastasis, nine of them are living.

In the statistical analysis, we stratified the cases according to genetic classification. We separated tumours known to have common specific chromosomal changes, e.g. synovial sarcomas, extraskeletal Ewing's sarcoma, myxoid liposarcoma and round cell liposarcoma. The remaining cases formed a mixed group, of which the largest proportion were pleomorphic sarcomas. Table 2 shows the stratification. Three sarcomas with specific chromosomal changes were included in the miscellaneous sarcoma group because they were single cases of alveolar rhabdomyosarcoma, extraskeletal myxoid chondrosarcoma and clear cell sarcoma (Fletcher, 1995; Sheer, 1997).

### Methods

Several different antibodies detect the same large nuclear active phase protein (Ki-67 antigen). In this study, we used the monoclonal Mib-1 antibody, which reacts with Ki-67 in formalin-fixed archival material.

The samples were deparaffinated in xylene and rehydrated in alcohol to distilled water. Antigen demasking was carried out by heating the samples in a microwave oven (850 W) in citric acid buffer (pH 6, 0.1 M) four times for 5 min. Metanol peroxidase (1.6%) was used to inhibit the endogenous peroxidase activity of the sample. Ki-67 staining was carried out by mouse anti-human monoclonal Mib-1 antibody (PharMingen). Our antibody dilution was 1:500, and we incubated samples overnight at room temperature. To reveal the binding of the primary antibody by peroxidase staining, we used the Vectastain ABC kit (Vector Laboratories). Haematoxylin was used for background staining.

When evaluating the results of the immunostaining, we chose the tumour area with the highest density of positive nuclear staining. A magnification of $10 \times 40$ was used. The percentage of positively stained nuclei was evaluated with an ocular grid of 100 ($10 \times 10$) squares. We counted all the positive nuclei from this area. To estimate the negative nuclei in the same area of 100 squares, we counted three different rows of ten squares and multiplied the mean of the results by ten. The percentage of positive nuclei was counted by dividing the positive stained cells by the entire number of cells in the same area. In the case of tumours with scarce cellularity, several fields were evaluated, and negative nuclei were counted from the whole grid area of 100 squares. All samples were scored by two independent observers (RH and TB). When the interobserver difference in the result was greater than 5%, the sample was rescored by the two investigators together.

A modification of the basic Hedley method was used in flow cytometric measurements (Hedley, 1989; Heiden et al, 1991). The sections (100 µm) from the paraffin blocks were placed in finemesh bags which were inserted into cassettes and then dewaxed and rehydrated in a tissue processor. Enzymatic digestion was carried out with Subtilisin Carlsberg (Sigma Protease type 24) and all centrifugation steps were omitted, resulting in nuclei suspensions with extremely low amounts of clumped nuclei and debris. The nuclei were stained with DAPI (final concentration 5 µM) and analysed using a PAS 2 flow cytometer. To confirm the ploidy of near-diploid populations, the DNA content of the identified nuclei was measured by means of a static image analysis system. Tumours were considered diploid if only one G1 peak was present. For determination of the DNA index in grossly aneuploid cell populations, the first peak to the left was assumed to represent diploid cells. For the determination of the percentage of cells in the S-phase of the cell cycle, the MultiCycle program with the sliced-nuclei option for background subtraction, developed by Rabinowich, was used (Phoenix Flow System, San Diego, CA, USA). Flow cytometry measurements were carried out at the Radiobiological Department of the Karolinska Institute in Stockholm (Huuhtanen et al, 1996).

### Statistical methods

Survival analyses were carried out with the Macintosh SPSS computer program, and all other analyses with the Macintosh Statistica program. The correlations between different variables, except ploidy, were calculated with the Spearman rank order correlation test. The confidence intervals of the Spearman rank

 © Cancer Research Campaign 1999

**Table 2** Pretreatment characteristics

| Characteristic | n | % |
|---|---|---|
| Sex | | |
| Female | 66 | 54 |
| Male | 57 | 46 |
| Age | | |
| Median (range), years | 53 (16–89) | |
| WHO performance status | | |
| 0 | 90 | 73 |
| 1 | 20 | 16 |
| 2 | 7 | 6 |
| 3 | 4 | 3 |
| Unknown | 2 | 2 |
| Location | | |
| Trunk | 26 | 21 |
| Limb | 97 | 79 |
| Site | | |
| Only cutaneous/subcutaneous | 39 | 32 |
| Deep | 82 | 67 |
| Unknown | 2 | 2 |
| Size | | |
| <5 cm | 34 | 28 |
| ≥5 cm | 86 | 70 |
| Unknown | 3 | 2 |
| Ploidy | | |
| Diploid | 59 | 48 |
| Non-diploid | 64 | 52 |
| Grade | | |
| I | 6 | 5 |
| II | 26 | 21 |
| III | 49 | 40 |
| IV | 42 | 34 |
| Histology | | |
| Malignant fibrous histiocytoma[a] (G2 = 4, G3 = 14, G4 = 16)[b] | 34 | 28 |
| Liposarcoma[c] (G1 = 2, G2 = 8, G3 = 2, G4 = 3) | 15 | 12 |
| Leiomyosarcoma (G1 = 2, G2 = 2, G3 = 5, G4 = 2) | 11 | 9 |
| Synovial sarcoma (G2 = 1, G3 = 9, G4 = 2) | 12 | 10 |
| Extraskeletal Ewing's sarcoma (G3 = 1, G4 = 7) | 8 | 6 |
| Fibrosarcoma (G1 = 1, G2 = 4, G3 = 2) | 7 | 6 |
| Malignant Schwannoma (G2 = 1, G3 = 2, G4 = 2) | 5 | 4 |
| Unclassified sarcoma (G1 = 1, G2 = 2, G3 = 8, G4 = 8) | 19 | 15 |
| Other sarcoma[d] (G2 = 4, G3 = 3, G4 = 3) | 12 | 10 |
| Histological grouping by known chromosomal changes | *n* (diploid *n*) | |
| Miscellaneous sarcoma | 94 (34) | 76 |
| Synovial sarcoma | 12 (11) | 10 |
| Myxoid liposarcoma and round cell liposarcoma | 9 (7) | 7 |
| Extraskeletal Ewing's sarcoma | 8 (7) | 7 |

[a]Storiform–pleomorphic *n* = 27, myxoid *n* = 7; [b]G1–G4 = number of patients with grade 1–4 tumours; [c]well-differentiated *n* = 2, myxoid *n* = 8, round cell *n* = 1, pleomorphic *n* = 3, pleomorphic–storiform *n* = 1; [d]myxofibrosarcoma *n* = 1, extraskeletal myxoid chondrosarcoma *n* = 1, clear cell sarcoma *n* = 1, alveolar rhabdomyosarcoma *n* = 1, malignant haemangiopericytoma *n* = 1, stromal sarcoma *n* = 1, angiosarcoma *n* = 2, epithelioid sarcoma *n* = 2, extraskeletal osteosarcoma *n* = 2.

order correlation test were calculated according to Altman (1994). The correlation between ploidy and Ki-67 was calculated with the Mann–Whitney *U*-test.

Analyses of disease-specific overall survival (DSOS) and metastasis-free survival (MFS) were carried out using the Cox regression model. In the Cox regression model analysis, Ki-67 and SPF were continuous variables. The prognostic value of the different cut-off points of Ki-67 and SPF was further investigated

**Table 3** Medians and ranges of Ki-67 and SPF of 123 tumours divided by histotype, grade and by genetic class

| Histotype | *n* | Ki-67 median (%) (range) | SPF median (%) (range) |
|---|---|---|---|
| Malignant fibrous histiocytoma | 34 | 26 (5.5–55) | 11.6 (0–38.2) |
| Liposarcoma | 15 | 4 (1–40) | 4.9 (0.9–24.3) |
| Leiomyosarcoma | 11 | 11 (5–35.5) | 10.3 (1–29.2) |
| Synovial sarcoma | 12 | 9.3 (3.5–20) | 4.0 (1.8–8.3) |
| Extraskeletal Ewing's sarcoma | 8 | 21 (7.5–44) | 9.3 (3.2–21.9) |
| Malignant schwannoma | 5 | 19 (9–34) | 6 (1.9–16.3) |
| Fibrosarcoma | 7 | 11.5 (3.5–34) | 4 (1–16.3) |
| Unclassified | 19 | 22 (8–48 | 11.2 (3.3–24.9) |
| Other | 12 | 18 (2–48) | 6.6 (1.7–15.3) |
| Grade (*n*) | | | |
| 1 | 6 | 6.5 (1–21) | 4.9 (1–24.2) |
| 2 | 26 | 11 (2–48) | 3.7 (0.9–18.5) |
| 3 | 49 | 18 (3–43) | 8.3 (0–29.5) |
| 4 | 42 | 28 (7.5–55) | 12.1 (1.2–38.2) |
| Histological grouping by known chromosomal changes | | | |
| Miscellaneous | 94 | 20.5 (1–55) | 9.7 (0–38.2) |
| Synovial sarcoma | 12 | 9.3 (3.5–20) | 4 (1.8–8.3) |
| Liposarcoma | 9 | 4 (2–32) | 2.4 (0.9–10.2) |
| Extraskeletal Ewing's sarcoma | 8 | 21 (7.5–35.5) | 9.25 (3.2–21.9) |

by dividing the material into two by 10% percentiles (i.e. cut-off points defining 10% of the patients in the low-risk and 90% in the high-risk group, 20% in the low-risk and 80% in the high-risk group, etc.). The corresponding cut-off values for Ki-67 and SPF are shown in Figure 1. For each cut-off point, the relative risk of death or distant relapse and 95% confidence levels were calculated by Cox regression analysis.

Survival curves were calculated by using the cut-point value of 10% for Ki-67, the value with the greatest discriminative power in the study of Choong et al (1994). Thirty-four (28%) patients had tumours with equal or lower Ki-67 value than 10%. In the group of miscellaneous sarcomas, 18 (19%) tumours of 94 cases were below this limit. To compare the results with SPF, we chose the cut-off point of 3.9% for SPF, which divided the material similarly to the Ki-67 cut-off point of 10% (i.e. 28% had equal or lower values of SPF). The survival curves have been calculated by the Kaplan–Meier method (Figures 2 and 3).

### RESULTS

The median value of Ki-67 was 18% (range 1–55%). The median of Ki-67 was highest in malignant fibrous histiocytomas and lowest in liposarcomas. Low-grade tumours had a lower median of Ki-67 than high-grade tumours. The medians and ranges of Ki-67 and SPF by grade, histotype and genetic class are shown in Table 3.

The Ki-67 staining score was strongly correlated with histological grade ($P < 0.001$), ploidy ($P < 0.001$) and SPF [$r_s$ (±95%CI) = 0.57 (0.44–0.68), $P < 0.001$]. There was no correlation between Ki-67 and tumour size ($P = 0.2$), or SPF and size ($P = 0.16$). SPF correlated with grade and ploidy ($P < 0.001$ both). The correlations between Ki-67 and SPF are shown in Figure 4, with diploid and non-diploid tumours presented separately.

© Cancer Research Campaign 1999 

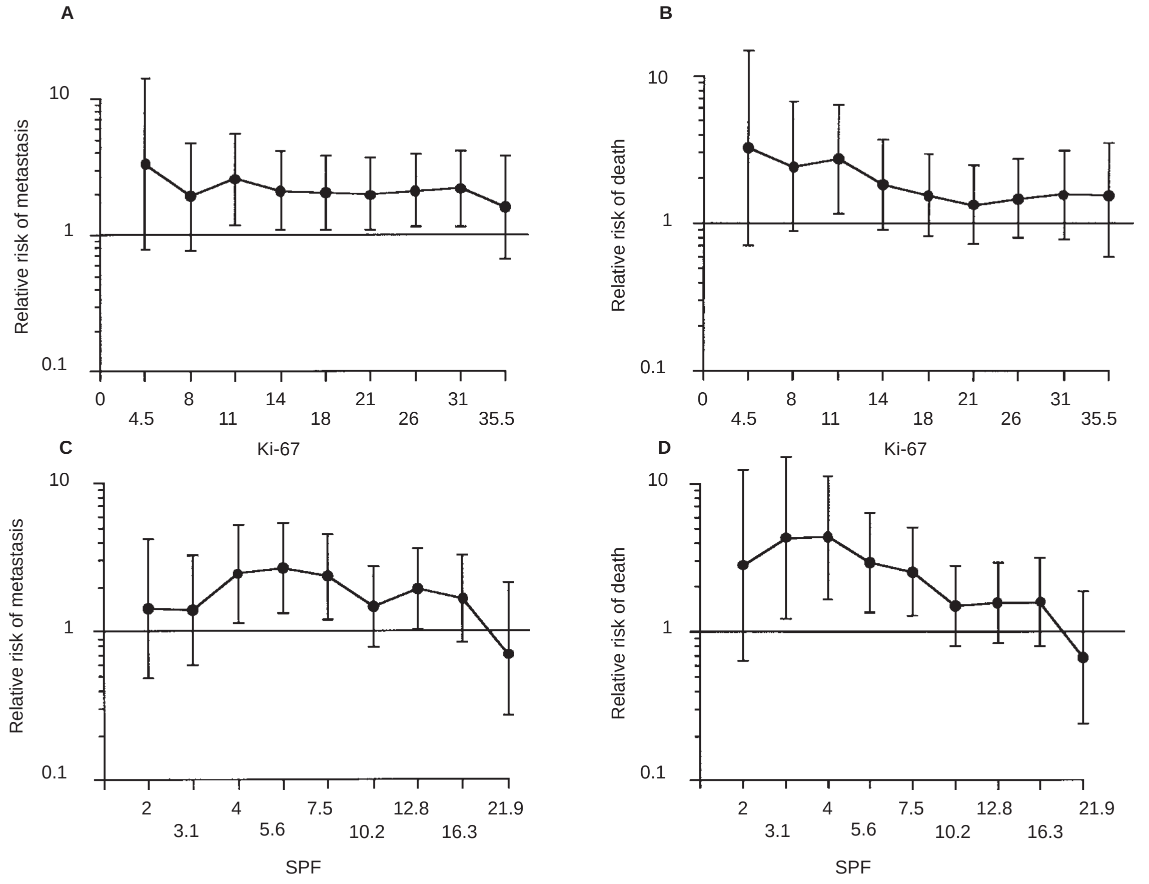

**Figure 1** Relative risks of metastases and death according to different cut-points of Ki-67 and SPF. 95% confidence intervals indicated by crossbars. (**A**) Metastasis-free survival (MFS) by Ki-67; (**B**) disease-free overall survival (DSOS) by Ki-67; (**C**) MFS by SPF; (**D**) DSOS by SPF

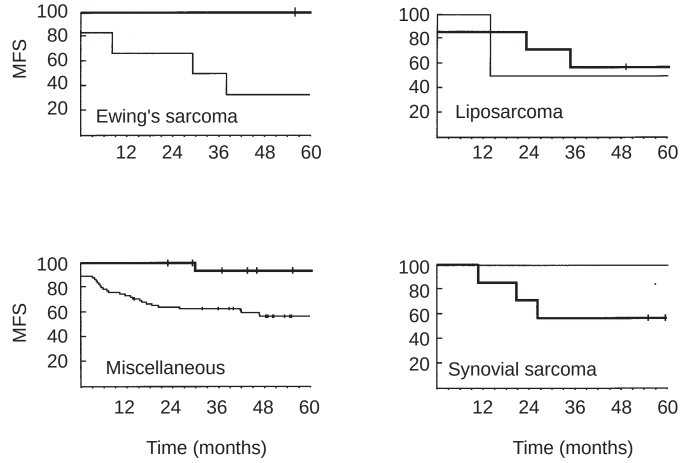

**Figure 2** Metastasis-free survival (MFS) in different genetic classes. Bold line Ki-67 ≤10%, fine line Ki-67 > 10%

When analysed as a continuous variable, Ki-67 was a stronger predictor of both MFS and DSOS ($P = 0.003$ and $P = 0.04$ respectively) than SPF ($P = 0.06$ and 0.07 respectively). When using cut-off points of 10% for Ki-67, it predicted MFS and DSOS ($P = 0.02$ and $P = 0.02$ respectively). When using a cut-off point of 3.9% for SPF, it predicted MFS and DSOS ($P = 0.03$ and $P = 0.003$ respectively). The estimated 5 years of MFS was 79% for tumours with Ki-67 ≤ 10% and 57% for tumours with Ki-67 > 10%. The estimated 5 years of DSOS was 82% for tumours with Ki-6 ≤ 10% and 60% for tumours with Ki-67 > 10%. The estimated 5 years of MFS was 81% for tumours with SPF ≤ 3.9% and 57% for tumours with SPF > 3.9%. The estimated 5 years of DSOS was 90% for tumours with SPF ≤ 3.9% and 57% for tumours with SPF > 3.9%. But when analysed separately in diploid and non-diploid tumour

 *© Cancer Research Campaign 1999*

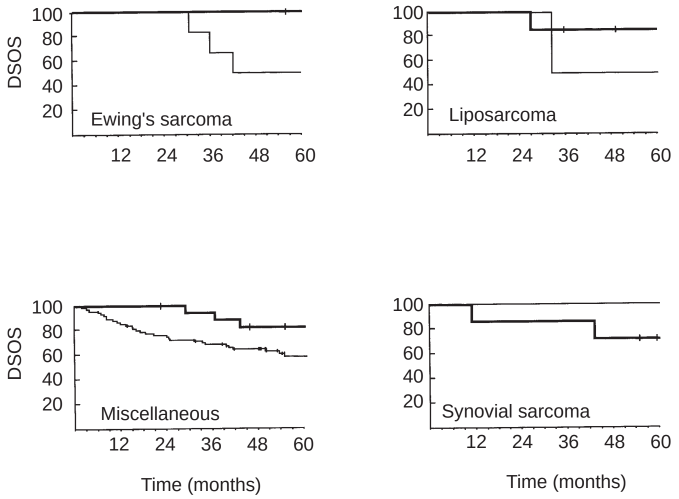

**Figure 3** Disease-specific overall survival (DSOS) in different genetic classes. Bold line Ki-67 ≤ %, fine line Ki-67 > 10%

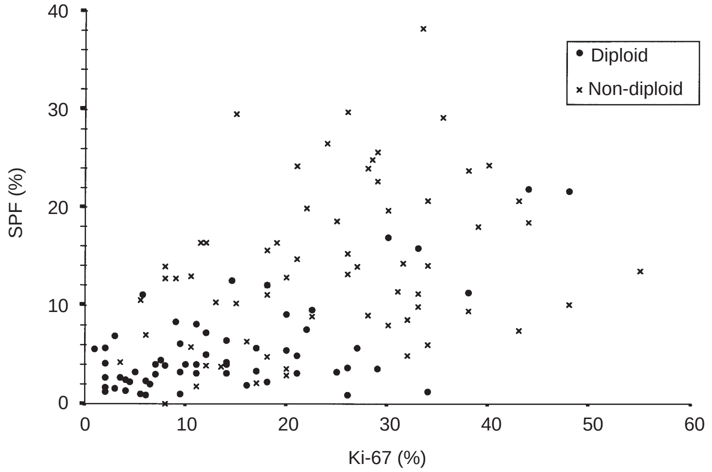

**Figure 4** Correlations between Ki-67 and SPF. Diploid and non-diploid tumours are represented separately

**Table 4** Ki-67 antigen expression and prognosis in STS

| Reference | *n* | Histology | Antibody | OS[a] *P*-value | Cut-off point (%) |
|---|---|---|---|---|---|
| Zehr et al (1990) | 29 | MFH[b] | Ki-67 | 0.43 | |
| Ueda et al (1989) | 34 | Mixed | Ki-67 | <0.005 | |
| Drobnjak et al (1994) | 172 | Mixed | Ki-67 | 0.03 | 20 |
| Choong et al (1994) | 182 | Mixed | Mib-1 | 0.0001 | 10 |
| Yang et al (1995) | 54 | MFH[b] | Mib-1 | 0.00008 | 25 |
| Levine et al (1997) | 52 | Mixed | Ki-67 | 0.0019 | 40 |
| Rudolph et al (1997) | 126 | Mixed | Ki-S11 | 0.0004 | 20 |
| | 125 | | Ki-S1 | 0.0008 | |
| Huuhtanen et al (1998) | 123 | Mixed | Mib-1 | 0.02[c] | 10 |

[a]OS, overall survival; [b]MFH, malignant fibrous histiocytoma; [c]DSOS disease-specific overall survival.

groups, SPF cut-point of 3.9 predicted DSOS only in diploid tumours and not in non-diploid tumours ($P$ = 0.02 and $P$ = 0.02 respectively). MFS and DSOS in the four genetic groups, according to the cut-off points of 10% for Ki-67 and 3.9% for the SPF, are shown in Figures 2 and 3. Analysed in this way, Ki-67 was of significant prognostic value only in the group of miscellaneous sarcomas ($P$-value for MFS <0.001 and $P$-value for DSOS = 0.01). Figure 4 gives the discriminative values of Ki-67 and SPF for MFS and DSOS as a function of various cut-off points by relative risk and confidence interval.

### DISCUSSION

The results of our study are in accordance with previous findings (Table 4), which indicate that Ki-67 is of prognostic value in STS. Compared with SPF, Ki-67 seemed to be a stronger prognostic factor. Ki-67 measurements are also more economical and easier to perform than SPF measurements. When comparing the discriminative values of different cut-off points, it became evident that the weaker prognostic effect of SPF seems to be due to the poor discriminative power of high SPF values.

With low cut-off points, SPF seemed to be an equally effective prognostic factor as Ki-67, which is in accordance with previous results (Collin et al, 1997; Gustafson et al, 1997). The results of Gustafson and of Collin are not directly comparable to ours, however, because both of these investigators excluded 35–37% of their tumours because of uninterpretable histograms. The majority of the excluded cases were non-diploid. Because of differences in techniques, we were able to calculate the SPF also in the majority of cases with non-diploid tumours. These histograms, nevertheless, did not add much to the prognostic value of SPF because SPF failed to be of prognostic value in non-diploid tumours, as shown previously (Huuhtanen et al, 1996).

Thus, in contrast to SPF, our results indicate that Ki-67 can be utilized to define a small group of patients with a high risk of relapse, whereas SPF and Ki-67 seemed equivalent in their ability to define a low-risk group. We can only speculate about why the highest SPF values fail to have prognostic implication. Because the SPF is a static rather than a dynamic proliferation parameter, a high value is not necessarily the result of a high proportion of cycling cells. A prolonged duration of the SPF, for example due to a highly disorganized genome, will also result in a high SPF. Our previously published result, i.e. that SPF is a statistically significant prognostic variable in diploid but not in non-diploid sarcomas, could thus support this hypothesis.

One difficulty in trying to find prognostic indicators for STS is the extreme heterogeneity of this group of tumours. Although many similarities in behaviour between different histotypes can be found, for example the propensity to blood-borne metastasis vs. lymphatic spread and the tendency to respect anatomical barriers in their growth, many entities still have distinct features. Especially sarcomas characterized by a specific pathognomonic gene translocation such as synovial sarcoma, Ewing's sarcoma, mesenchymal chondrosarcoma and myxoid liposarcoma often display specific behaviour. Features distinguishing these entities from other STS include frequent late relapse in synovial sarcoma, myxoid liposarcoma and Ewing's sarcoma, soft-tissue metastases in myxoid liposarcoma and bone metastases in Ewing's sarcoma (Enzinger and Weiss, 1995).

In this study, we therefore decided to stratify the cases with respect to histogenesis, with special reference to disease-specific chromosomal translocations. When we analysed the prognostic value of proliferation measured by Ki-67 in these subgroups, it appeared that the discriminative power is quite strong in the mixed group of miscellaneous, mostly pleomorphic sarcomas. There was no clear indication of prognostic value in any of the tumours characterized by specific gene translocations because these tumour groups were too small to be analysed separately. Probably, the unexpected result in the synovial sarcoma patient group, in which low proliferative activity correlated with poorer outcome, is simply coincidental. In the group of miscellaneous pleomorphic sarcomas, however, the results seem sufficiently promising to justify a large confirmatory study.

### ACKNOWLEDGEMENTS

This study was supported by grants from the Finnish Cancer Foundation, Finska Läkaresällskapet (Finnish Medical Society), the Albin Johansson Foundation, and by a state subsidy for research and development at Helsinki University Central Hospital. We thank Ms Pirjo Jutila for technical assistance.

### REFERENCES

Altman D (1994) Relation between two continuous variables. In *Practical Statistics for Medical Research*, pp. 293–294. Chapman and Hall: London

Camplejohn RS, Ash CM, Gillett CE et al (1995) The prognostic significance of DNA flow cytometry in breast cancer: results from 881 patients treated in a single centre. *Br J Cancer* **71**: 140–145

Choong PF, Akerman M, Willen H et al (1994) Prognostic value of Ki-67 expression in 182 soft tissue sarcomas. Proliferation – a marker of metastasis? *APMIS* **102**: 915–924

Collin F, Chassevent A, Bonichon F et al (1997) Flow cytometric DNA content analysis of 185 soft tissue neoplasms indicates that S-phase fraction is prognostic factor for sarcomas. French Federation of Cancer Centers (FNCLCC) Sarcoma Group. *Cancer* **79**: 2371–2379

Dettmar P, Harbeck N, Thomssen C et al (1997) Prognostic impact of proliferation-associated factors MIB1 (Ki-67) and S-phase in node-negative breast cancer. *Br J Cancer* **75**: 1525–1533

Drobnjak M, Latres E, Pollack D et al (1994) Prognostic implications of p53 nuclear overexpression and high proliferation index of Ki-67 in adult soft-tissue sarcomas. *J Natl Cancer Inst* **86**: 549–554

Enzinger J and Weiss S (1995) *Soft Tissue Tumors*. Mosby Year-Book: St. Louis

Fletcher J (1995) Cytogenetic analysis of soft tissue tumors. In *Soft Tissue Tumors*. Enzinger F and Weiss S (eds.), pp. 105–118. Mosby-Year Book: St. Louis

Gasparini G, Boracchi P, Verderio P and Bevilacqua P (1994) Cell kinetics in human breast cancer: comparison between the prognostic value of the cytofluorimetric S-phase fraction and that of the antibodies to Ki-67 and PCNA antigens detected by immunocytochemistry. *Int J Cancer* **57**: 822–829

Gerdes J, Li L, Schlueter C et al (1991) Immunobiochemical and molecular biologic characterization of the cell proliferation-associated nuclear antigen that is defined by monoclonal antibody Ki-67. *Am J Pathol* **138**: 867–873

Gustafson P, Ferno M, Akerman M et al (1997) Flow cytometric S-phase fraction in soft-tissue sarcoma: prognostic importance analysed in 160 patients. *Br J Cancer* **75**: 94–100

Hedley DW (1989) Flow cytometry using paraffin-embedded tissue: five years on. *Cytometry* **10**: 229–241

Heiden T, Wang N and Tribukait B (1991) An improved Hedley method for preparation of paraffin-embedded tissues for flow cytometric analysis of ploidy and S-phase. *Cytometry* **12**: 614–621

Huuhtanen RL, Blomqvist CP, Wiklund TA et al (1996) S-phase fraction of 155 soft tissue sarcomas: correlation with clinical outcome. *Cancer* **77**: 1815–1822

Joensuu H, Toikkanen S and Klemi PJ (1990) DNA index and S-phase fraction and their combination as prognostic factors in operable ductal breast carcinoma. *Cancer* **66**: 331–340

Keshgegian AA and Cnaan A (1995) Proliferation markers in breast carcinoma. Mitotic figure count, S-phase fraction, proliferating cell nuclear antigen, Ki-67 and MIB-1. *Am J Clin Pathol* **104**: 42–49

Levine EA, Holzmayer T, Bacus S et al (1997) Evaluation of newer prognostic markers for adult soft tissue sarcomas. *J Clin Oncol* **15**: 3249–3257

Molino A, Micciolo R, Turazza M et al (1997). Ki-67 immunostaining in 322 primary breast cancers: associations with clinical and pathological variables and prognosis. *Int J Cancer* **74**: 433–437

Pierga JY, Leroyer A, Viehl P et al (1996) Long term prognostic value of growth fraction determination by Ki-67 immunostaining in primary operable breast cancer. *Breast Cancer Res Treat* **37**: 57–64

Railo M, Nordling S, von Boguslawsky K et al (1993) Prognostic value of Ki-67 immunolabelling in primary operable breast cancer. *Br J Cancer* **68**: 579–583

Railo M, Lundin J, Haglund C et al (1997) Ki-67, p53, ER-receptors, ploidy and S-phase as prognostic factors in T1 node negative breast cancer. *Acta Oncol* **36**: 369–374

Rudolph P, Kellner U, Chassevent A et al (1997) Prognostic relevance of a novel proliferation marker, ki-s11, for soft-tissue sarcoma – a multivariate study. *Am J Pathol* **150**: 1997–2007

 © Cancer Research Campaign 1999

Sheer D, (1997) Chromosomes in solid tumors. In: *Introduction to the Cellular and Molecular Biology of Cancer*. Franks LM and Teich NM (eds.), pp. 217–220. Oxford University Press: London

Sigurdsson H, Baldetorp B, Borg A et al (1990) Flow cytometry in primary breast cancer: improving the prognostic value of the fraction of cells in the S-phase by optimal categorisation of cut-off levels (see comments). *Br J Cancer* **62**: 786–790

Ueda T, Aozasa K, Tsujimoto M et al (1989). Prognostic significance of Ki-67 reactivity in soft tissue sarcomas. *Cancer* **63**: 1607–1116

Veronese SM, Gambacorta M, Gottardi O et al (1993) Proliferation index as a prognostic marker in breast cancer. *Cancer* **71**: 3926–3931

Weikel W, Beck T, Mitze M and Knapstein PG (1991) Immunohistochemical evaluation of growth fractions in human breast cancers using monoclonal antibody Ki-67. *Breast Cancer Res Treat* **18**: 149–154

Weikel W, Brumm C, Wilkens C, Beck T and Knapstein PG (1995). Growth fractions (Ki-67) in primary breast cancers, with particular reference to node-negative tumors. *Cancer Detect Prev* **19**: 446–450

Wiklund T, Huuhtanen R, Blomqvist C et al (1996) The importance of a multidisciplinary group in the treatment of soft tissue sarcomas. *Eur J Cancer* **32(A)**: 269–273

Yang P, Hirose T, Hasegawa T et al (1995) Prognostic implication of the p53 protein and Ki-67 antigen immunohistochemistry in malignant fibrous histiocytoma. *Cancer* **76**: 618–625

Zehr RJ, Bauer TW, Marks KE and Weltevreden A (1990) Ki-67 and grading of malignant fibrous histiocytomas. *Cancer* **66**: 1984–1990

*© Cancer Research Campaign 1999*
